# Evaluation of *Bacillus subtilis* ATCC PTA-122264 on the fecal characteristics and microbiota of healthy adult dogs subjected to an abrupt diet change

**DOI:** 10.3389/fvets.2025.1617072

**Published:** 2025-07-17

**Authors:** Patrícia M. Oba, Olivia R. Swanson, Yifei Kang, Julio C. Mioto, John F. Menton, Elena Vinay, Mathieu Millette, Melissa R. Kelly, Kelly S. Swanson

**Affiliations:** ^1^Department of Animal Sciences, University of Illinois Urbana-Champaign, Urbana, IL, United States; ^2^Roy J. Carver Biotechnology Center, University of Illinois, Urbana, IL, United States; ^3^Kerry Group, Beloit, WI, United States; ^4^Kerry (Canada), Laval, QC, Canada; ^5^Science Made Simple, LLC, Winston Salem, NC, United States; ^6^College of Veterinary Medicine, University of Illinois Urbana-Champaign, Urbana, IL, United States; ^7^Division of Nutritional Sciences, University of Illinois Urbana-Champaign, Urbana, IL, United States

**Keywords:** canine microbiota, canine nutrition, dietary transition, probiotic, biotics

## Abstract

Abrupt dietary transitions are common in pets, but can lead to digestive disturbances, altered gut microbiota composition, and impaired intestinal integrity. The consumption of live microorganisms may have potential to mitigate these effects by stabilizing the gut microbiota and enhancing intestinal functionality. The current study aimed to evaluate the effects of *Bacillus subtilis* ATCC PTA-122264 supplementation on fecal characteristics, microbiota composition, and dysbiosis index of dogs undergoing an abrupt dietary change. Twelve healthy adult spayed female beagle dogs (6.0 ± 1.14 yr; 8.7 ± 0.91 kg body weight) were used in a replicated 3 × 3 Latin square design. In each experimental period, dogs were allotted to one of three treatments and fed a high-fiber kibble diet for 28 d: (1) 250 mg/d of maltodextrin (control), (2) 1 × 10^9^ colony-forming units (CFU)/d of *B. subtilis*, or (3) 5 × 10^9^ CFU/d of *B. subtilis*. All dogs were then abruptly transitioned to a high-protein, high-fat canned diet and fed for 14 d. Fresh fecal samples were collected before (d 0) and 2, 6, 10, and 14 d after the diet change for fecal scoring, pH, dry matter (DM) content, and microbiota analysis. Data were statistically analyzed to identify differences due to treatment, time, and treatment*time interactions, with *p* < 0.05 accepted as being significant. Diet change did not impact fecal pH or scores but reduced fecal DM percentage and bacterial alpha diversity measures. Bacterial beta diversity analysis revealed a distinct shift in the microbial community following the diet transition. Diet change reduced (*p* < 0.05) the abundances of short-chain fatty acid (SCFA)-producing bacteria and increased (*p* < 0.05) the relative abundance of potentially pathogenic bacteria, resulting in an elevated (*p* < 0.05) dysbiosis index. *B. subtilis* supplementation did not attenuate the microbial shifts caused by the diet transition. These findings confirm that an abrupt diet change significantly impacts some stool characteristics and fecal microbiota populations of dogs. Further investigation of *Bacillus* spp. strains and dosages is required to determine the potential benefits that they may provide during dietary transition.

## Introduction

Diet exerts a significant influence on the gastrointestinal (GI) health of dogs, modulating fecal microbial populations and metabolite concentrations. Abrupt dietary change, which is common in dogs, has been associated with digestive disturbances, GI discomfort, and loose stools (1, 2). Furthermore, such dietary changes can adversely affect intestinal integrity, alter gut microbiota composition, and disrupt the production of fermentation end-products (1, 3–5). In general, significant changes in the gut microbiota occur within a few days following a dietary change. In dogs fed different diets, microbiota shifts have been observed as early as 2 d after an abrupt diet change, with stability typically achieved within 6 to 10 d (5).

Probiotic studies with companion animals have demonstrated their ability to mitigate GI disorders triggered by dietary changes. A study evaluating *Bacillus subtilis* C-3102 in dogs demonstrated that *B. subtilis*-treated dogs had firmer feces, higher fecal scores, and lower fecal ammonia concentrations than control dogs (6). Dietary supplementation with *B. subtilis* C-3102 has also been shown to improve fecal quality, increase nutrient digestibility, and positively shift gut health parameters by reducing ammonia concentrations, increasing fecal SCFA concentrations, and increasing fecal *Lactobacillus* spp. and enterococci (7). Similarly, *Saccharomyces cerevisiae* CNCM I-5660 supplementation was shown to reduce fecal pH and biogenic amine, ammonia, and aromatic compound concentrations, while increasing fecal butyrate concentrations and lowering the dysbiosis index (DI) of dogs following an abrupt dietary change when compared with controls (3). Collectively, those studies suggest that the consumption of live microorganisms may enhance intestinal functionality in dogs and may provide benefits during dietary transition.

Although probiotics are recognized for their potential health benefits, there is a lack of studies evaluating the specific effects of *B. subtilis* ATCC PTA-122264 on gut microbiota, dysbiosis index, and fecal characteristics in dogs subjected to abrupt dietary change. In a study recently conducted by our team, *B. subtilis* ATCC PTA-122264 supplementation reduced the relative abundances of *Streptococcus*, *Escherichia coli*, and *Blautia* in dogs, indicating its potential to modulate the gut microbiota (8). Those findings indicate that *B. subtilis* may improve stool quality, reduce fecal odor, and positively modulate the gut microbiota of dogs. Given that *B. subtilis*-based probiotics may have the capacity to positively influence intestinal and microbial function, they may aid during dietary transition. Therefore, the objective of the current study was to assess the fecal characteristics and microbiota of *B. subtilis*-supplemented dogs undergoing an abrupt dietary change. We hypothesized that dogs supplemented with *B. subtilis* would have a more stable gut microbiome and greater ability to adapt to a new diet.

## Materials and methods

All animal procedures were approved by the University of Illinois Institutional Animal Care and Use Committee prior to experimentation (protocol #22217).

### Animals, treatments and diets

A replicated 3 × 3 Latin square design experiment was conducted. Twelve healthy adult spayed female beagle dogs (6.0 ± 1.14 yr old; 8.7 ± 0.91 kg) were used. All dogs were housed in an environmentally controlled facility at the University of Illinois Urbana-Champaign. Dogs always had free access to fresh water. Based on previous feeding records, dogs were fed once daily (8–9 am) to maintain body weight. Dogs were weighed and body condition was assessed (9) once a week prior to feeding.

The following treatments were tested: 250 mg/d of maltodextrin (control); 1 × 10^9^ colony-forming units (CFU)/d of *B. subtilis* ATCC PTA-122264 (Low dose; Kerry, Inc., Beloit, WI); and 5 × 10^9^ CFU/d of *B. subtilis* ATCC PTA-122264 (High dose). Maltodextrin and *B. subtilis* treatments were provided prior to each meal with gelatin capsules before each meal. During the first 28 d of each experimental period, dogs were allotted to one of the three treatments and fed a dry extruded kibble commercial diet formulated to meet all Association of American Feed Control Officials nutrient recommendations for adult dogs at maintenance (10) and containing no probiotics or prebiotics and little fermentable fiber (Best Dog 21/12; Mid-South Feeds Inc., Alma, GA). At that time, all dogs were then abruptly changed to a wet canned diet (Pedigree Chopped Ground Dinner with Chicken Adult Canned Wet Dog Food; Mars Petcare US, Franklin, TN) formulated to meet all Association of American Feed Control Officials nutrient recommendations for adult dogs at maintenance (10) and fed for 14 d. Analyzed chemical composition of diets is listed in Table 1.

**Table 1:** Analyzed chemical and energy composition of diets.

Item	Dry^a^	Wet^b^
Dry matter, %	88.42	23.55
Dry matter basis		
Organic matter	91.55	87.64
Ash	8.45	12.36
Crude protein	22.95	43.74
Acid-hydrolyzed fat	13.81	24.63
Total dietary fiber	22.75	16.10
Insoluble fiber	20.63	11.82
Soluble fiber	2.12	4.28
Gross energy, kcal/g	5.00	5.61

a Dry: Best Dog 21/12, Mid-South Feeds Inc., Alma, GA. Ingredients: Ground yellow corn, chicken byproduct meal, pork meat and bone meal, wheat middlings, poultry fat (preserved with mixed tocopherols), salt, calcium propionate, potassium chloride, artificial garlic flavoring, calcium carbonate, vitamin E (as D-alpha tocopheryl acetate), riboflavin supplement, niacin supplement, biotin, calcium pantothenate, vitamin A supplement, menadione sodium bisulfite complex (source of vitamin K activity), thiamine mononitrate (source of vitamin B1), pyridoxine hydrochloride (source of vitamin B6), vitamin B12 supplement, vitamin D3 supplement, ferrous sulfate, zinc sulfate, zinc oxide, manganese sulfate, copper sulfate, sodium selenite, calcium iodate, cobalt carbonate, folic acid, mineral oil.

b Wet: Pedigree Chopped Ground Dinner with Chicken Adult Canned Wet Dog Food, Mars Petcare US, Franklin, TN. Ingredients: Chicken, sufficient water for processing, meat by-products, animal liver, brewers rice, wheat flour, minerals (potassium chloride, magnesium proteinate, zinc sulfate, selenium, copper proteinate, manganese sulfate, copper sulfate, potassium iodide), carrageenan, sodium tripolyphosphate, dried yam, xanthan gum, vitamins (choline chloride, vitamin E supplement, thiamine mononitrate, calcium pantothenate, biotin, riboflavin supplement, vitamin A supplement, vitamin D3 supplement, vitamin B12 supplement), natural flavor, guar gum, yellow #6, yellow #5.

### Fecal collection, scoring, and handling

Fresh (within 15 min of defecation) fecal samples were collected prior to (d 0) and 2, 6, 10, and 14 d after diet change for scoring and the measurement of pH, dry matter (DM) percentage, and microbiota. First, fecal samples were scored using the following scale: 1 = hard, dry pellets, small hard mass; 2 = hard, formed, dry stool; remains firm and soft; 3 = soft, formed, and moist stool, retains shape; 4 = soft, unformed stool, assumes shape of container; and 5 = watery, liquid that can be poured. Fecal pH was measured immediately using an AP10 pH meter (Denver Instrument, Bohemia, NY) equipped with a Beckman Electrode (Beckman Instruments Inc., Fullerton, CA). After pH was measured, an aliquot was collected for DM determination in accordance with AOAC (11) procedures using a 105°C oven. An aliquot of fresh feces was then transferred to sterile cryogenic vials (Nalgene, Rochester, NY), placed on dry ice, and then stored at −80°C until microbiota analysis.

### Chemical analyses

Diet and fecal samples were analyzed for DM and ash according to the Association of Official Analytical Chemists (11); (methods 934.01 and 942.05), with organic matter calculated. Crude protein was calculated from Leco (TruMac N, Leco Corporation, St. Joseph, MI) total nitrogen values according to AOAC (11). Total lipid content (acid-hydrolyzed fat) was determined according to the methods of the American Association of Cereal Chemists (12) and Budde (13). Total dietary fiber of diets was determined according to Prosky et al. (14). Gross energy was measured using an oxygen bomb calorimeter (Model 6200, Parr Instruments, Moline, IL).

### Fecal DNA extraction and PacBio sequencing of 16S rRNA gene amplicons

Total DNA from fecal samples was extracted using Mo-Bio PowerSoil kits (MO BIO Laboratories, Inc., Carlsbad, CA). Concentrations of extracted DNA were quantified using a Qubit 3.0 Fluorometer (Life Technologies, Grand Island, NY). The quality of extracted DNA was assessed by electrophoresis using agarose gels (E-Gel EX Gel 1%; Invitrogen, Carlsbad, CA). The Roy J. Carver Biotechnology Center at the University of Illinois performed PacBio sequencing. The 16S amplicons were generated with the barcoded full-length 16S primers from PacBio and the 2× Roche KAPA HiFi Hot Start Ready Mix (Roche, Wilmington, MA). Full-length 16S PacBio (Pacific Biology, Menlo Park, CA) primers (forward: AGRGTTYGATYMTGGCTCAG; reverse: RGYTACCTTGTTACGACTT) were added in accordance with the PacBio protocol. The amplicons were pooled and converted to a library with the SMRT Bell Express Template Prep kit 3.0 (Pacific Biology, Menlo Park, CA). The library was sequenced on a SMRT cell 8 M in the PacBio Sequel IIe using the CCS sequencing mode and a 15-h movie time. Analysis of CCS was done using SMRT Link V11.1.0 using the following parameters: minimum passes 3, and minimum rq 0.999; HiFi presets (minimum score of 80; minimum end score of 50, minimum reference (read) span of 0.75); asymmetric (different, minimum number of scoring barcode regions 2).

### Microbial data analysis

PacBio-based FASTQ reads were processed using a Nextflow-based workflow and analyzed with DADA2 v1.22 (15) for trimming, denoising, and generating amplicon sequence variants (ASV). Taxonomic classification was performed using the Ribosomal Database Project classifier implemented in DADA2 (36) with the SILVA 138.1 database formatted for PacBio HiFi reads.^1^ Multiple sequence alignment and phylogenetic analysis were conducted using DECIPHER v2.22 (37) and FastTree v2.1.10 (38). Quality filtering retained sequences with a minimum quality score of 20, and samples were rarefied to 49,463 reads. The taxonomic classifications produced by DADA2, as well as their quantifications, were imported into phyloseq (version 1.44.0) in R (version 4.3.1). The rarefied samples were used for alpha and beta diversity analysis. Principal coordinate analysis was performed using weighted and unweighted unique fraction metric (UniFrac) distances. Analysis of compositions of microbiomes with bias correction (ANCOMBC) was estimated using the ANCOMBC package (version 2.4.0) to determine specific taxa that were statistically responsible for the observed discrimination between treatment and period, with Benjamini–Hochberg adjusted *p*-value, and *q* < 0.05 was accepted as statistically significant. Spearman’s rank correlation coefficient (*r*) carried out using microbiome package (version 1.24.0), with Benjamini–Hochberg adjusted *p*-value, and *q* < 0.05 was accepted as statistically significant.

### Quantitative PCR and dysbiosis index

DNA was extracted from an aliquot of 100–120 mg fecal sample using a bead-beating method with a MoBio Power soil DNA isolation kit. qPCR assays were used to quantify total bacteria, *Blautia*, *Clostridium* (*Peptacetobacter*) *hiranonis*, *Escherichia coli*, *Faecalibacterium*, *Fusobacterium*, *Streptococcus*, and *Turicibacter* according to (39). Both positive and negative controls were included for all qPCR assays to ensure the accuracy and reliability of the results. The DI was calculated based on the results of the qPCR assays using a previously described algorithm (39).

### Statistical analysis

Data were analyzed using the Mixed Models procedure of SAS version 9.4 (SAS Institute, Inc., Cary, NC). Treatment and day were considered fixed effects, while dog was considered a random effect. Data were tested for normality using the UNIVARIATE procedure of SAS. Differences between treatment, day, and treatment*day interactions were determined using repeated measures and a Fisher-protected least significant difference test with a Tukey adjustment to control for experiment-wise error. A probability of *p* < 0.05 was accepted as being statistically significant. Reported pooled standard errors of the means were determined according to the Mixed Models procedure of SAS.

## Results

**Figure 1:**
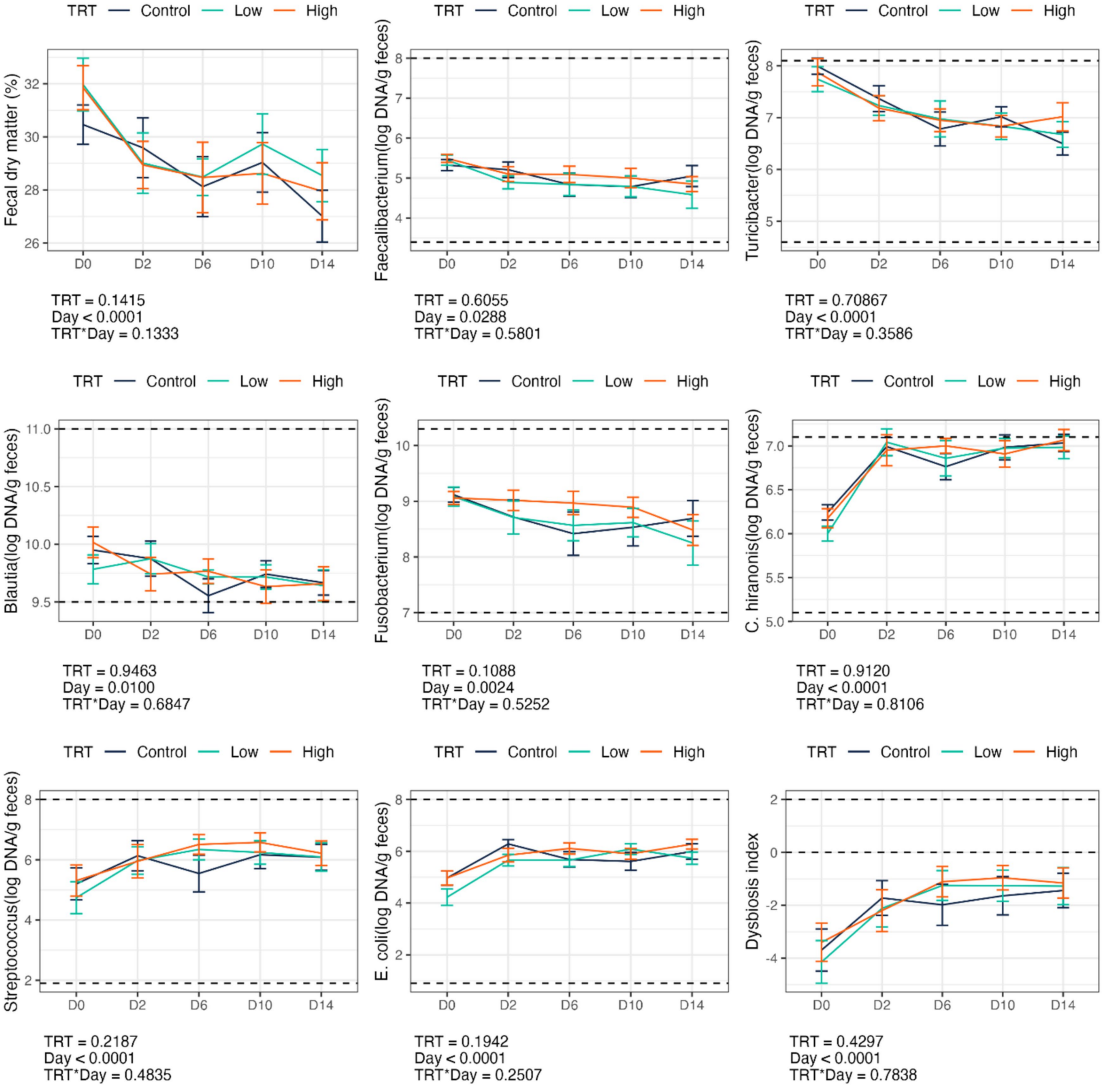
Fecal dry matter, bacterial abundance (log DNA/g feces), and dysbiosis index of dogs supplemented with a *B. subtilis* probiotic before and after an abrupt diet change. D0, before abrupt dietary change; D2, 2 days after abrupt dietary change; D6, 6 days after abrupt dietary change; D10, 10 days after abrupt dietary change; D14, 14 days after abrupt dietary change.

Fecal scores and pH were not affected by *B. subtilis* supplementation or dietary change (Table 2). However, the abrupt dietary change reduced (*p* < 0.0001) fecal DM % and increased (*p* < 0.0001) fecal dysbiosis index (Figure 1). The dietary change also increased (*p* < 0.0001) fecal *Streptococcus*, *E. coli*, and *C. hiranonis* abundances and decreased (*p* < 0.01) fecal *Faecalibacterium*, *Turicibacter*, *Blautia*, and *Fusobacterium* abundances (Figure 1). Diet-induced changes to fecal characteristics, DI, and bacterial abundances were not impacted by treatment (i.e., *B. subtilis* supplementation) or treatment*day interactions (Figure 1).

**Table 2:** Fecal characteristics of dogs supplemented with a *B. subtilis* probiotic before and after an abrupt diet change.

Item	D0^a^			D2			D6			D10			D14					*p*-value	
	Con^b^	Low	High	Con	Low	High	Con	Low	High	Con	Low	High	Con	Low	High	SEM	TR	D	D*T
Score^c^	3.00	2.63	2.75	2.50	2.99	2.63	2.54	2.58	2.71	2.67	2.38	2.63	2.71	2.63	2.67	0.17	0.25	0.33	0.44
pH	7.11	7.12	6.99	6.90	9.08	7.05	7.09	7.10	6.98	7.02	7.09	7.18	7.05	7.09	7.25	0.46	0.42	0.92	0.18

a D0, before abrupt dietary change; D2, 2 days after abrupt dietary change; D6, 6 days after abrupt dietary change; D10, 10 days after abrupt dietary change; D14, 14 days after abrupt dietary change.

b Con, 250 mg/d of maltodextrin (control); low dose (Low), 1 × 10^9^ CFU/d of *B. subtilis*; high dose (High), 5 × 10^9^ CFU/d of *B. subtilis*; SEM, pooled standard error of the means; TR, treatment effect; D, day effect; D*T, treatment*day interaction effect.

c Fecal score: 1 = hard, dry pellets, small hard mass; 2 = hard formed, dry stool, remains firm and soft; 3 = soft, formed and moist stool, retains shape; 4 = soft, unformed stool, assumes shape of container; 5 = watery, liquid that can be poured.

**Figure 2:**
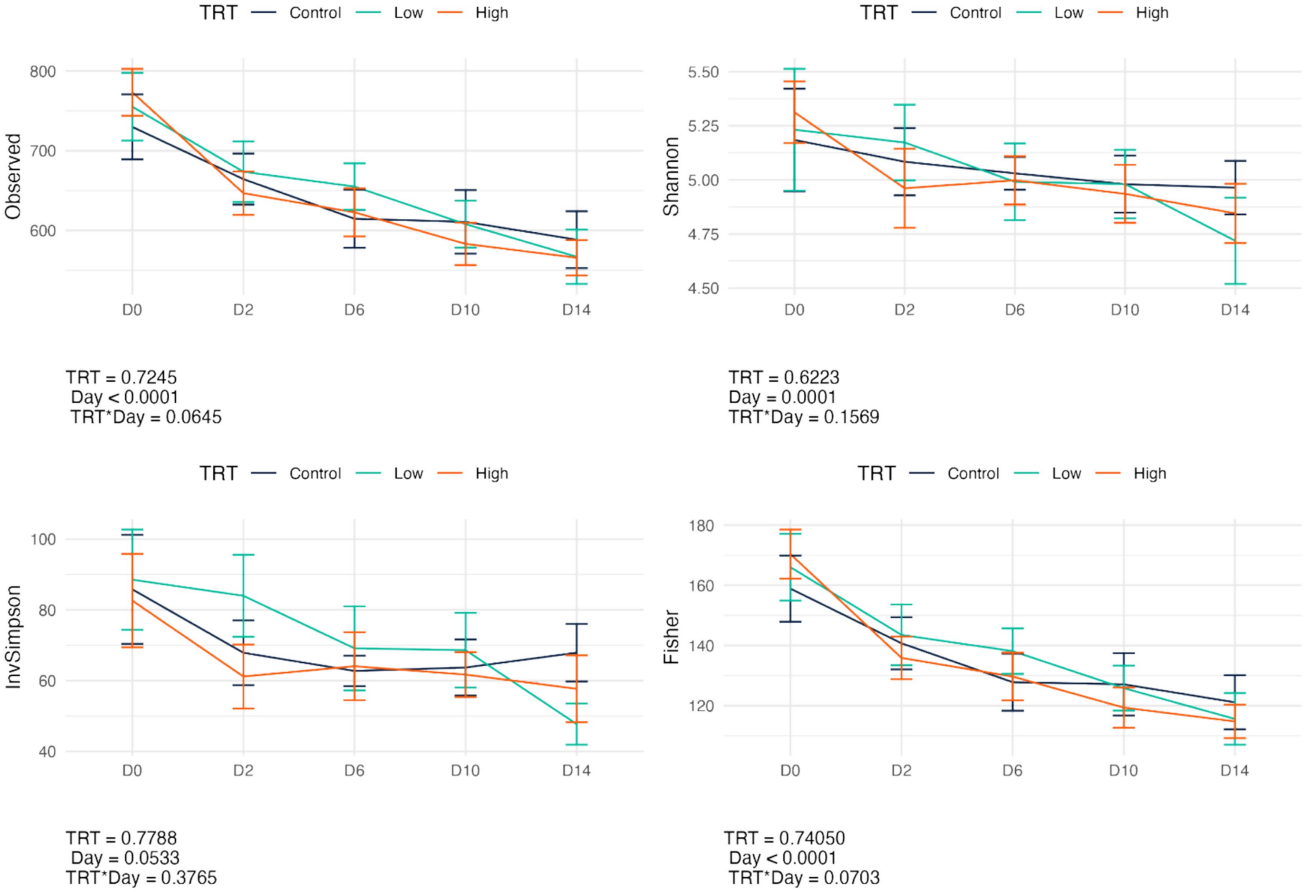
Change in bacterial alpha diversity measures (Observed, Observed ASV; Shannon, Shannon Index; InvSimpson, Inverse Simpson Index; Fisher, Fisher index) of fecal samples from dogs supplemented with *B. subtilis* before and after an abrupt diet change. D0, before abrupt dietary change; D2, 2 days after abrupt dietary change; D6, 6 days after abrupt dietary change; D10, 10 days after abrupt dietary change; D14, 14 days after abrupt dietary change.

**Figure 3:**
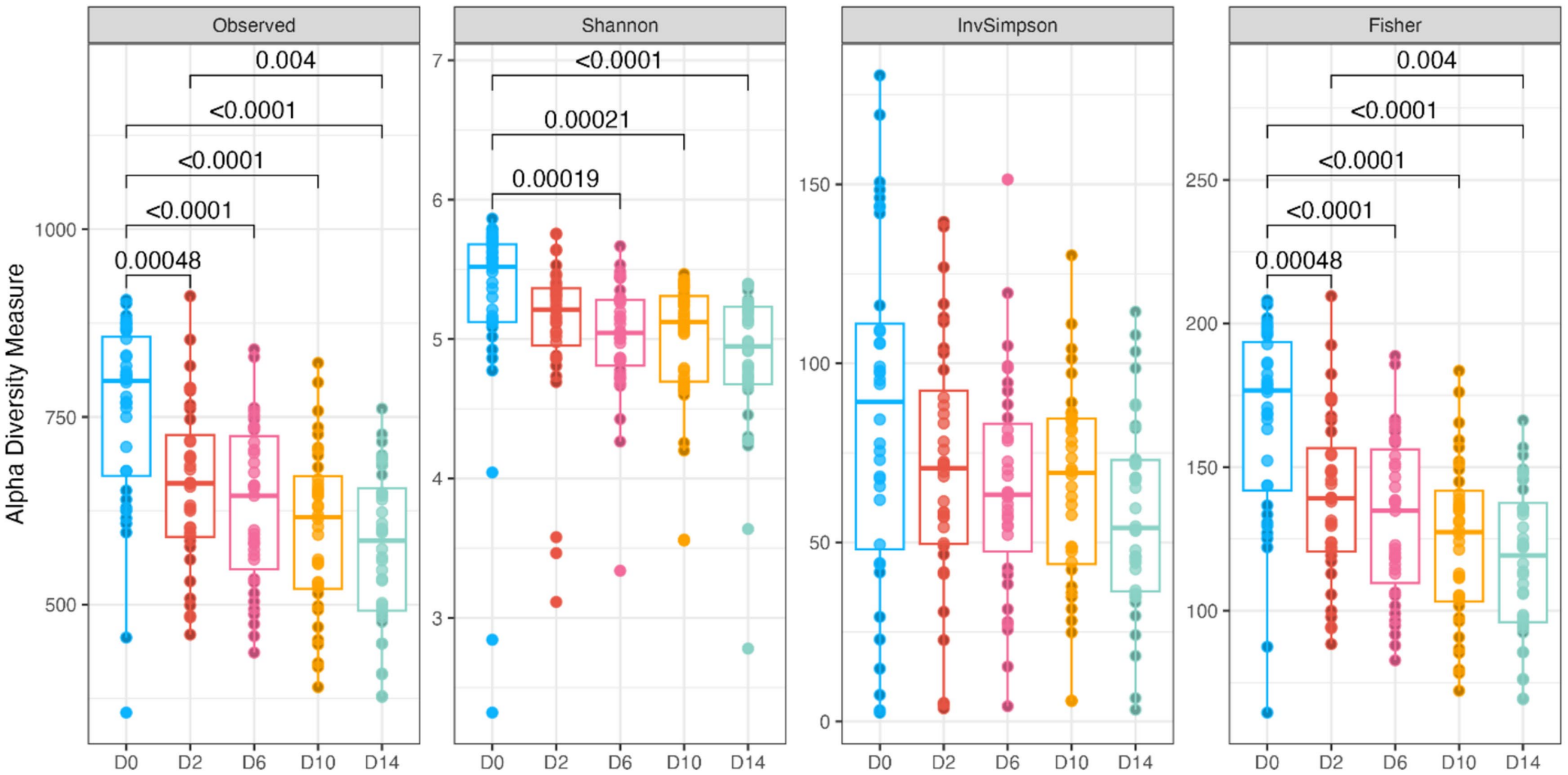
Bacterial alpha diversity measures (Observed, Observed ASV; Shannon, Shannon Index; InvSimpson, Inverse Simpson Index; Fisher, Fisher index) of fecal samples from dogs were affected by diet change. D0, before abrupt dietary change; D2, 2 days after abrupt dietary change; D6, 6 days after abrupt dietary change; D10, 10 days after abrupt dietary change; D14, 14 days after abrupt dietary change.

**Figure 4:**
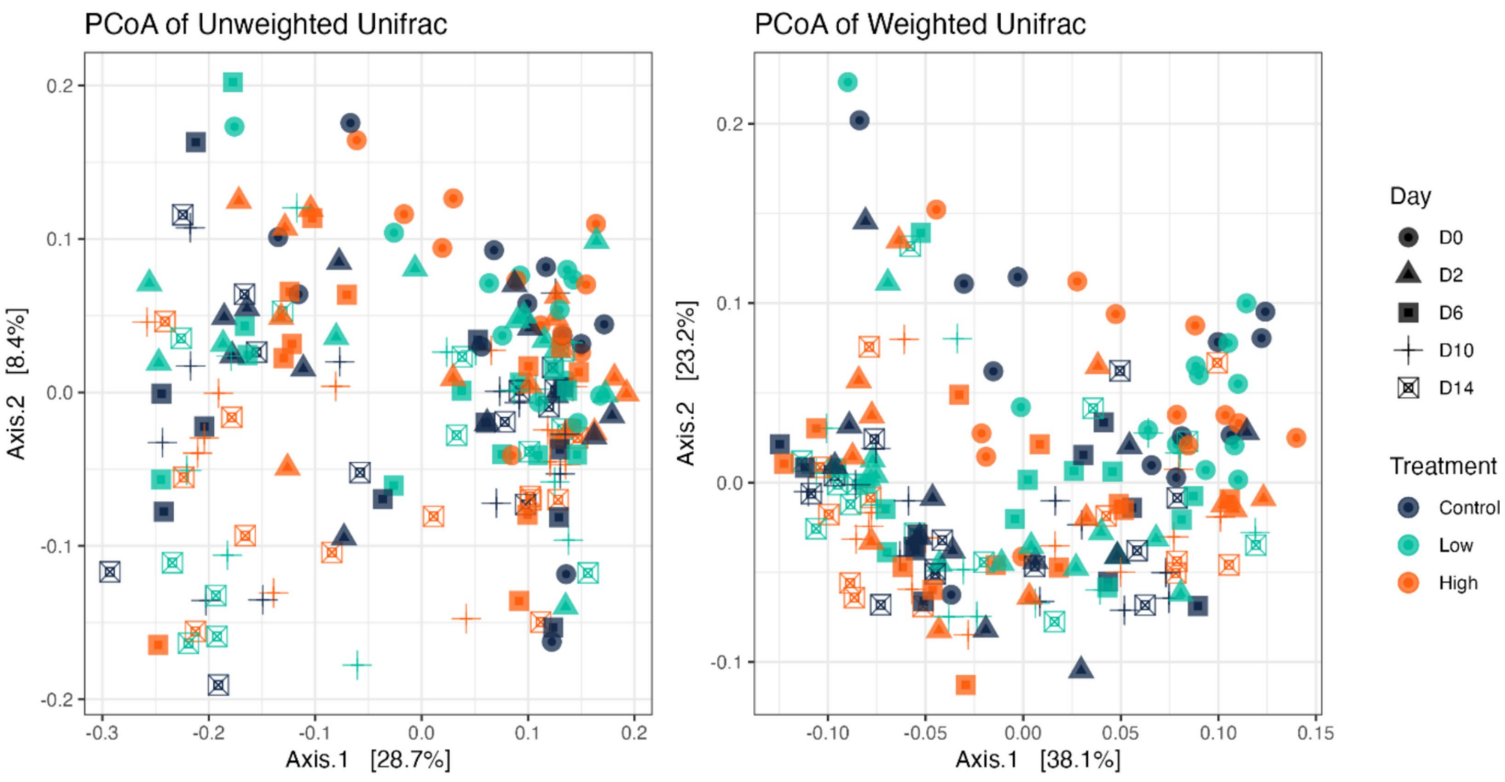
Bacterial beta diversity indices of fecal samples from dogs supplemented with *B. subtilis* before and after an abrupt diet change, as assessed by unweighted and weighted UniFrac distances. D0, before abrupt dietary change; D2, 2 days after abrupt dietary change; D6, 6 days after abrupt dietary change; D10, 10 days after abrupt dietary change; D14, 14 days after abrupt dietary change.

**Figure 5:**
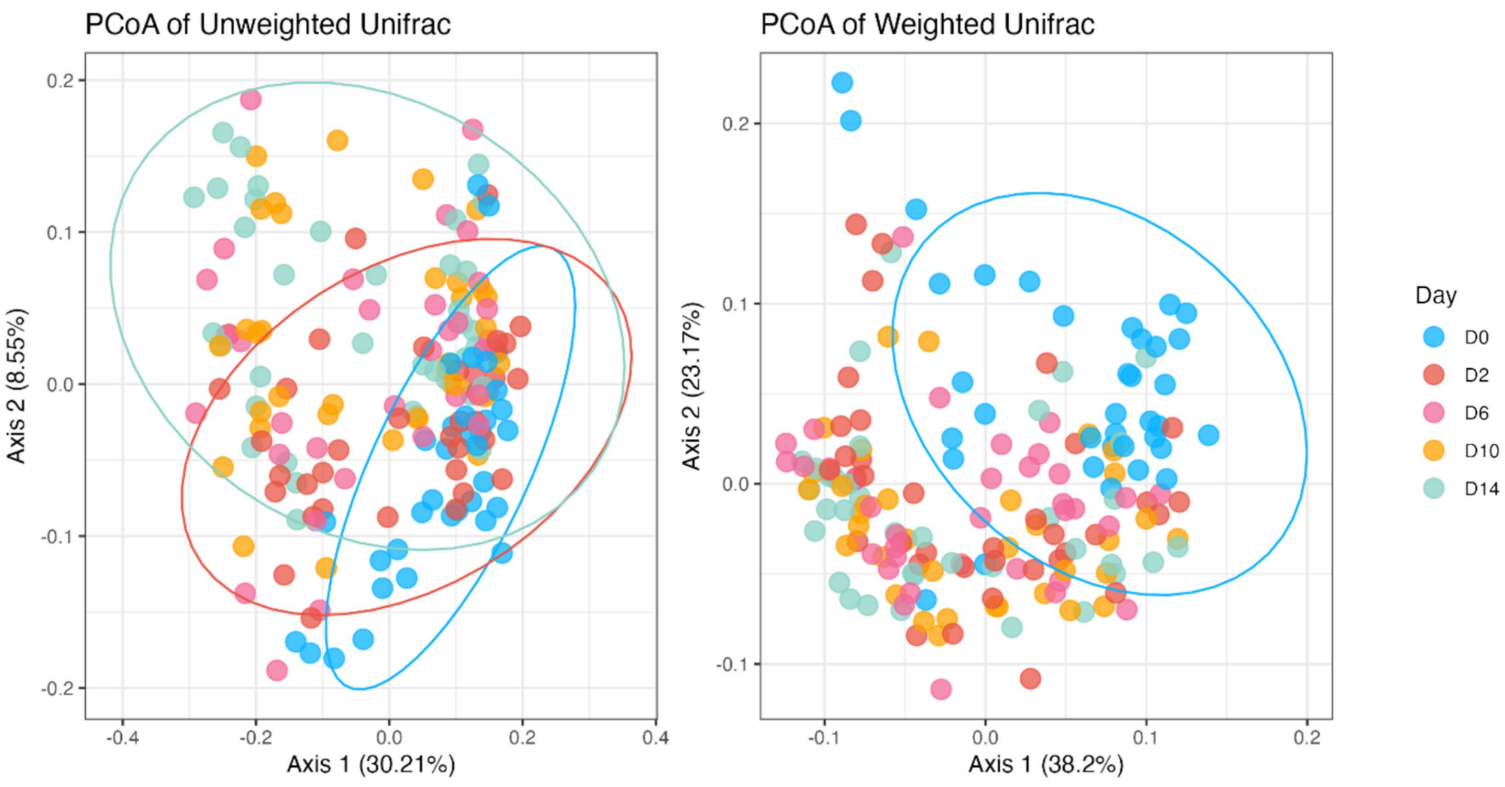
Bacterial beta diversity indices of fecal samples from dogs before and after an abrupt dietary change, as assessed by unweighted and weighted UniFrac distances. D0, before abrupt dietary change; D2, 2 days after abrupt dietary change; D6, 6 days after abrupt dietary change; D10, 10 days after abrupt dietary change; D14, 14 days after abrupt dietary change.

Fecal bacterial alpha diversity measures were reduced (*p* < 0.05) with diet change (day effect) but were not affected by *B. subtilis* supplementation or treatment*day interactions (Figures 2, 3). Three of the four alpha diversity measures were affected, with observed ASV, Fisher Index, and the Shannon Index being reduced (*p* < 0.005) by diet change. Beta diversity, as assessed by unweighted and weighted UniFrac distances, was also affected by diet change (Figures 4, 5). The principal coordinate analysis plots of weighted and unweighted UniFrac distances show separation of microbial communities, with samples shifting after dietary change. Samples collected prior to dietary change were different (*p* < 0.05) than samples collected at all other time points. Moreover, samples collected 2 d after dietary change were different (*p* < 0.05) than those collected 14 d after dietary change.

**Figure 6:**
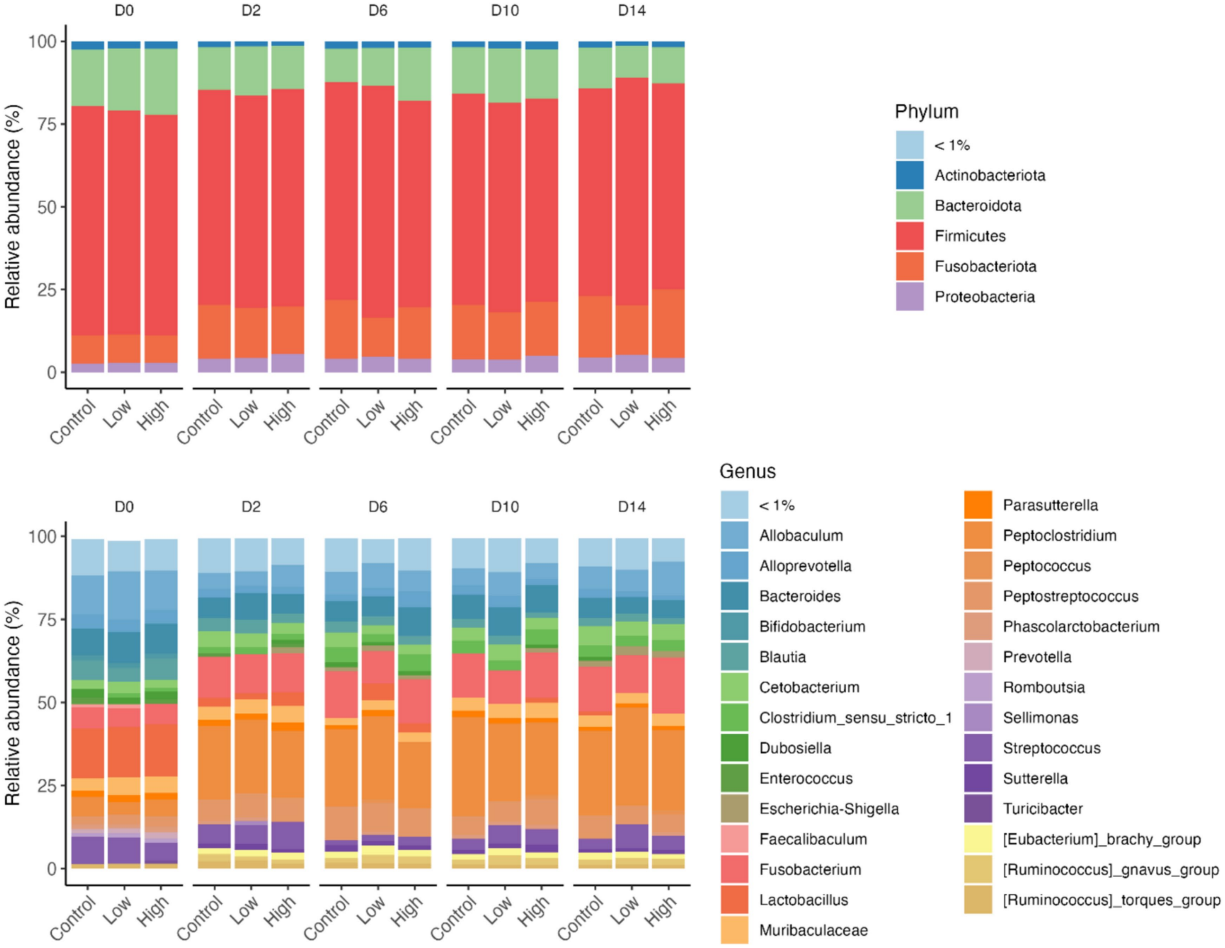
Relative abundance of bacteria phyla and genus of fecal samples from dogs supplemented with *B. subtilis* before and after an abrupt diet change. Only abundant taxa are shown; phyla and genera in low relative abundances (<1% in all samples) were combined into “<1%.” D0, before abrupt dietary change; D2, 2 days after abrupt dietary change; D6, 6 days after abrupt dietary change; D10, 10 days after abrupt dietary change; D14, 14 days after abrupt dietary change.

**Figure 7:**
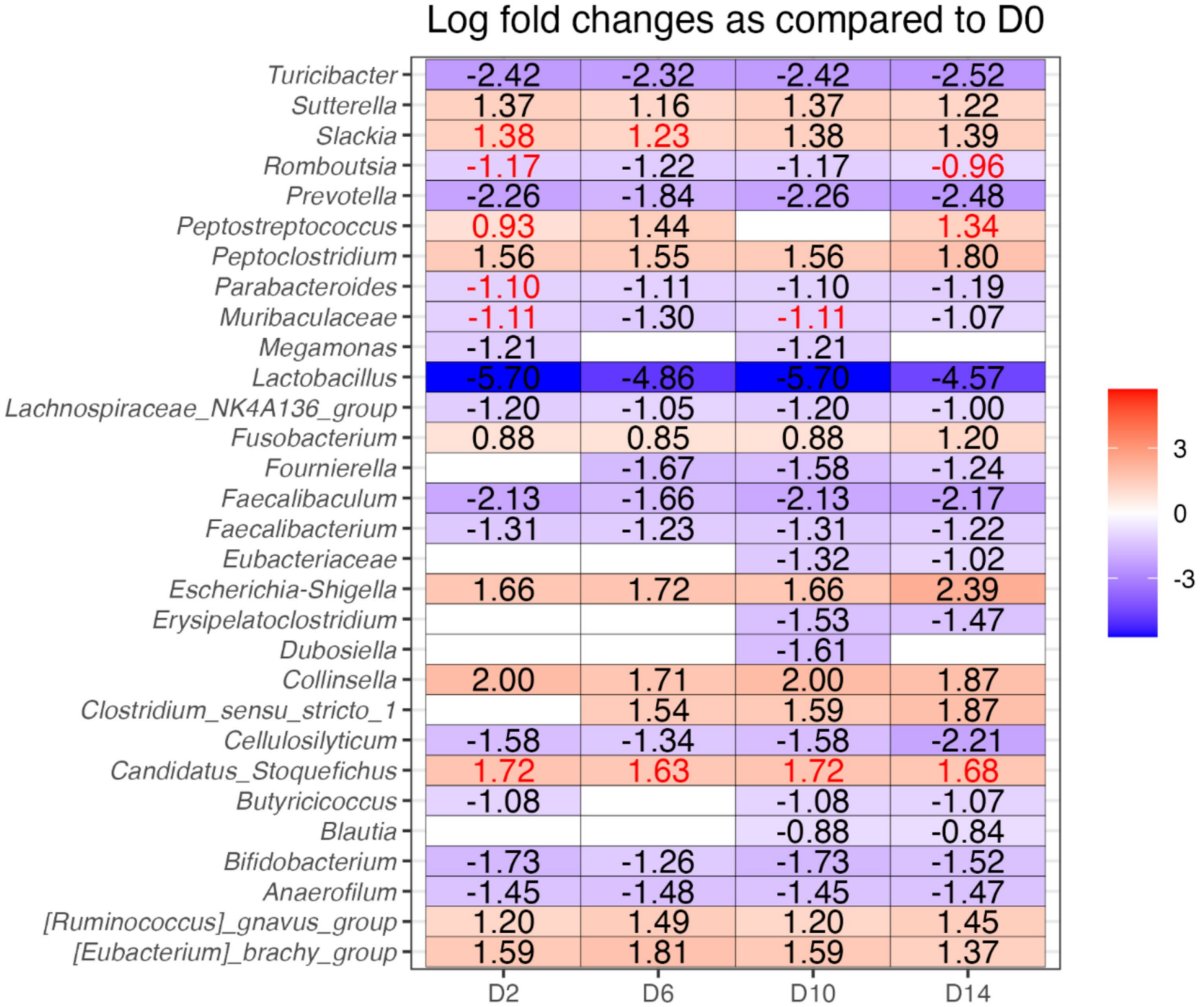
Analysis of composition of microbiomes with bias correction (ANCOMBC), illustrating which bacterial genera were differentially abundant between fecal samples of dogs before and after dietary change (difference greater than 1 and *q* < 0.05). D0, before abrupt dietary change; D2, 2 days after abrupt dietary change; D6, 6 days after abrupt dietary change; D10, 10 days after abrupt dietary change; D14, 14 days after abrupt dietary change. The values in red text did not pass the sensitivity analysis for pseudo-count addition.

The relative abundances of several bacterial phyla and genera were affected by diet change, but not by *B. subtilis* supplementation or treatment*day interactions (Figures 6, 7). Using ANCOMBC analysis, the relative abundances of fecal *Turicibacter*, *Prevotella*, *Lactobacillus*, *Lachanospiraceae NK4A136 group*, *Faecalibaculum*, *Faecalibacterium*, *Cellulosilyticum*, *Bifidobacterium*, and *Anaerofilum* were reduced (*p* < 0.05) with the dietary change. In contrast, the relative abundances of fecal *Sutterella*, *Peptoclostridium*, *Fusobacterium*, *Escherichia-Shigella*, *Collinsella*, *Ruminococcus gnavus group*, and *Eubacterium brachy group* were increased (*p* < 0.05) with the dietary change.

## Discussion

Abrupt dietary change is common in dogs and can lead to GI disturbances, including altered fecal consistency and fecal microbiota and metabolite shifts (1–5). While the gut microbiota adapt within a few days following a diet transition, the extent and stability of these changes depend on various factors, including microbiota balance and the presence of beneficial taxa (3, 4). Live microorganisms, such as *B. subtilis*, have been studied for their role in modulating gut microbiota, enhancing fecal characteristics, and mitigating digestive disturbances (6, 7). However, the specific effects of *B. subtilis* ATCC PTA-122264 on microbiome stability, dysbiosis index, and fecal characteristics in dogs undergoing abrupt dietary transitions remain unexplored. This study evaluated whether supplementation with *B. subtilis* ATCC PTA-122264 could promote a more stable gut microbiome and improve fecal characteristics in dogs following an abrupt diet change.

Consistent with previous research, diets with markedly different ingredient compositions, macronutrient profiles, and formats (wet vs. dry) were selected to facilitate shifts in the microbiome. Dogs were moved from a dry extruded kibble diet containing moderate protein and fat concentrations and high dietary fiber concentrations to a canned diet containing higher protein and fat concentrations and lower dietary fiber concentrations. The fiber type was also different with twice the amount of soluble fiber present in the canned diet. Diet transition immediately reduced fecal DM but was stabilized after 6 days. The diet change did not impact fecal scores or pH, however. Previous studies have shown that fecal scores (increased, indicating looser stools) and fecal DM (decreased) shifted and stabilized within 2 days of a dietary change (5), while fecal pH also decreased following the transition and reached stability within the same period (3).

Compared with the control, supplementation with *B. subtilis* ATCC PTA-122264 did not affect fecal scores, DM, or pH after the dietary change. These results were similar to the supplementation of *S. cerevisiae* (0.12 g per dog per day) in a previous study, where fecal pH, scores, or DM were similar to control animals following a dietary transition (3). Without the challenge of dietary transition, previous studies have reported that *B. subtilis* C-3102 supplementation at 1 × 10^9^ CFU/d and 2 × 10^9^ CFU/d led to firmer stools, higher fecal DM, and lower fecal pH than control dogs (7). Another study demonstrated that dietary supplementation with 0.01% *B. subtilis* C-3102 (1 × 10^1^⁰ CFU/g) resulted in drier feces and higher fecal scores than those observed in control dogs (6). In our previous study testing *B. subtilis* ATCC PTA-122264 at doses of up to 5 × 10^9^ CFU/day, fecal characteristics were not affected but there was a tendency for reduced apparent total tract digestibility of DM, organic matter, and energy (8). While fecal DI tended to be affected, supplementation resulted in lower abundances of *Streptococcus*, *E. coli*, and *Blautia* in dogs receiving the low treatment (1 × 10^9^ CFU/day), suggesting potential microbiota-modulating effects (8). Those findings highlight strain-dependent differences in *B. subtilis* effects on fecal characteristics and microbiota modulation, suggesting that the abrupt dietary transition in this study may have presented a greater challenge to GI stability than *B. subtilis* supplementation alone could mitigate.

Transitioning from the high-fiber kibble diet to the high-protein, high-fat canned diet led to a reduction in SCFA-producing bacteria (e.g., *Faecalibacterium*, *Turicibacter*, *Blautia*, *Fusobacterium*) and an increase in potentially pathogenic bacteria (e.g., *E. coli*, *C. hiranonis*, and *Streptococcus*), resulting in a higher DI. Similarly, dogs fed a high-protein (44%, DM basis), high-fat (28%, DM basis) bones and raw food diet had a greater abundance of *E. coli*, *Streptococcus*, and *C. perfringens*, as well as a higher DI, than dogs fed commercially available kibble (31% crude protein and 18% fat, DM basis). Additionally, fecal *Faecalibacterium* abundance was lower in dogs consuming the bones and raw food diet than those fed kibble (16). In a previous study evaluating *S. cerevisiae* during an abrupt dietary change, dogs in the treatment group demonstrated a lower DI, lower *E. coli* abundance, and higher *Turicibacter* abundance than control animals, regardless of the day. Furthermore, transitioning from a low-protein (21%, DM basis), low-fiber (6%, DM basis) diet to a high-protein (28%, DM basis), high-fiber (27%, DM basis) diet improved (reduced) the DI, increased fecal *Fusobacterium* abundance, and decreased fecal *C. hiranonis* and *Streptococcus abundances* (3). Those findings suggest that dietary fiber content plays a significant role in microbiota modulation and that specific probiotic strains may offer benefits during dietary transition.

The ANCOMBC analysis in the current study revealed a reduction in SCFA-producing genera (e.g., *Turicibacter*, *Prevotella*, *Lactobacillus*, Lachanospiraceae NK4A136 group, *Faecalibaculum*, *Faecalibacterium*, *Cellulosilyticum*, *Bifidobacterium*, *Anaerofilum*) and an increase in potentially pathogenic bacteria (e.g., *Peptoclostridium*, *Escherichia-Shigella*, *Ruminococcus gnavus group*) following the abrupt dietary change. These findings are consistent with prior research showing that fiber intake in dogs promotes the growth of beneficial SCFA-producing bacteria, such as *Turicibacter*, *Lactobacillus*, *Lachnospira*, *Faecalibacterium*, *Bifidobacterium* and decrease *Escherichia* and *Peptoclostridium* (17–19). SCFA-producing genera, including *Bifidobacterium*, *Lactobacillus*, *Faecalibacterium*, *Anaerofilum*, *Prevotella*, Lachnospiraceae NK4A136 group, and *Faecalibaculum* utilize dietary fibers as substrates, contributing to GI health (19–25). Additionally, *Cellulosilyticum* has been identified as a bacterium capable of breaking down both fiber and protein, highlighting its potential role in microbial metabolism (26). High-protein diets often increase fecal *Peptoclostridium*, *E. coli*, and *Streptococcus*, decrease fecal *Bifidobacterium* and *Faecalibacterium*, increase concentrations of fecal protein catabolites (e.g., phenols, indoles, and branched-chain fatty acids), and reduce fecal concentrations of SCFA (16, 27–31). Moreover, *C. perfringens*, *E. coli*, and *Streptococcus* are recognized as potential pathogens (32–34), while *Ruminococcus gnavus group* has been positively associated with parvovirus GI infections in dogs (35).

Abrupt dietary changes in dogs, particularly those involving significant shifts in fiber, protein, and fat content, can lead to notable alterations in GI function, including changes in fecal characteristics and disruptions to the gut microbiota. While the gut microbiota has the capacity to adapt to dietary transitions, the extent of this adaptability can vary based on factors such as the presence of beneficial microbes. In this study, *B. subtilis* ATCC PTA-122264 supplementation was not able to attenuate the changes to fecal characteristics or microbiota. Other studies have shown positive effects of different probiotic strains, including *Bacillus* species, under stable conditions, suggesting that further exploration of different doses or strains may reveal more pronounced benefits. Nevertheless, the dietary change in this study led to an increase in potentially pathogenic bacteria and a decrease in beneficial SCFA-producing genera, which is consistent with previous research. This highlights the complexity of the relationship between diet and the gut microbiome and suggests that further investigation into the effects of different *Bacillus* strains and dietary interventions is necessary to better understand their role in maintaining GI health during abrupt diet changes in dogs.

## Data Availability

The raw sequencing data are available in the NCBI Sequence Read Archive (SRA) under BioProject PRJNA1277247. The data are publicly available.

## Footnotes

^1^ https://zenodo.org/record/4587955
